# Effect of slow-release nitrogenous fertilizers on dry matter accumulation, grain nutritional quality, water productivity and wheat yield under an arid environment

**DOI:** 10.1038/s41598-022-18867-5

**Published:** 2022-08-30

**Authors:** Iqra Ghafoor, Muhammad Habib ur Rahman, Muhammad Usama Hasnain, Rao Muhammad Ikram, Mahmood Alam Khan, Rashid Iqbal, Muhammad Iftikhar Hussain, Ayman EL Sabagh

**Affiliations:** 1 Department of Agronomy, MNS University of Agriculture Multan, Punjab, Pakistan; 2 Institute of Crop Science and Resource Conservation (INRES), Crop Science, University of Bonn, 53115 Bonn, Germany; 3 Institute of Plant Breeding and Biotechnology, MNS-University of Agriculture, Multan, Pakistan; 4 Department of Agronomy, The Islamia University, Bahawalpur, 63100 Punjab Pakistan; 5 Department of Plant Biology and Soil Science, Universidad de Vigo, Campus Lagoas Marcosende, 36310, Vigo, Spain; 6 Department of Field Crops, Faculty of Agriculture, Siirt University, Siirt, Turkey

**Keywords:** Plant sciences, Environmental sciences

## Abstract

Slow release nitrogenous fertilizers can improve crops production and reduce the environmental challenges in agro-ecosystem. There is a need to test the efficiency and performance under arid climatic conditions. The study investigates the effect of slow-release fertilizers (urea, neem coated urea (NCU), sulfur coated urea (SCU) and bioactive sulfur coated urea (BSCU)) on the growth, productivity and grain nutritional qualities of wheat crop. Slow-release fertilizers (SRF) with nitrogen levels (130,117,104 and 94 kg ha^−1^) were applied with equal splits at sowing, 20 and 60 days after sowing (DAS). Research showed that the BSCU with 130 kg ha^−1^ increased dry matter accumulation (1989 kg ha^−1^) after anthesis and grain yield 4463 kg ha^−1^. The higher plant height (102 cm) was attained by 130 kg N ha^−1^ SCU while the minimum (77.67 cm) recorded for 94 kg N ha^−1^ as urea source. Maximum grain NPK concentrations (3.54, 0.66 and 1.07%) were recorded by BSCU 130 kg N ha^−1^ application. While, the minimum NPK (0.77, 0.19 and 0.35%) were observed by Urea 94 kg N ha^−1^. The high irrigation water use efficiency (WUE) recorded (20.92 kg ha^−1^ mm^−1^) and a crop index of 25.52% by BSCU 130 kg N ha^−1^ application. Research findings show that generally all SRF but particularly BSCU proved effective and can be recommended for wheat crop under arid environment.

## Introduction

Commercial fertilizers fulfill the global food demand about 40–60%. Nitrogenous and phosphoric fertilizers application is generally higher due to basic need of the cereal's growth and development^1–3^. The nitrogen use efficiencies (NUE) and phosphorus use^2^ efficiencies (PUE) range between 30 to 40% and 5 to 25% only for crop production, respectively^1,4^. The nitrogen is a basic element that fuels the crop growth and development, uptake of K and P, yield and grain quality^2,5^. Like N, potassium (K) and phosphorus (P) are also considered as macronutrients and have different important role in crop growth against abiotic stresses^2,6–8^. The NUE and PUE show the plant’s ability for N and P uptake and available N and P in the crop seeds^9,10^. The NUE is currently less than 50% for cereals including wheat due to higher nitrogen needs for production^2,11^. The soil nitrogen fertilizers application is sensitive and must meet crop’s requirements^10,12^. Likewise, nitrogen, potassium (K) and phosphorus (P) are also have different important roles in crop growth against different abiotic stresses^6,7^. The phosphorus involves in different growth processes like seedling, root growth, maturity, and seed quality. It is also important for seed formation, water use efficiency and root growth in plants. The potassium maintains cell turgidity by osmoregulation and stomatal regulation in plants^3,13^. The K activates more than seventy types of enzymes. The potassium uptake and phosphorus depend on plant nitrogen utilization and its availability in the soil^2,13^. The N, P and K regulate the utilization of each other nutrient due to synergistic effects in crop plants^8^. The fate of K and P depend on soil type and adsorption capacity as well. The 57% of applied K adsorbed on soil particles, based on the quantity and soil fertility^14^.

Excessive inorganic fertilizers application causes environmental pollution and deteriorates the groundwater quality as well^2,15^. Non-recommended and injudicious use of NPK based fertilizers cause seedling burning, lodging, disturb soil fauna, and decreased soil fertility and finally resulting in low crop yield. The two important concepts about nitrogen assimilation indicate that when plant requires nitrogen, soil has readily N to supply the plants immediately^16^. Improper nitrogen use also causes economic losses and leads to environmental pollution^15^. Crusciol et al.^17^ showed that recommended NPK application minimized losses and improve yield in wheat crop. The recommended NPK fertilizers enhance protein contents and grain weight, fertilizer use efficiencies and wheat yield^18,19^. To enhance the NPK efficiency and the production, the 4R principles (right amount, the right time, right place, and right source) are advised to approve for fertilizers^20^. The law of diminishing return also showed that constantly enhanced fertilizer application will not be an acceptable technique in augmenting crop production^21^.

The fertilizer's use efficiencies can be enhance by balancing, maintenance and fertilizer qualities. Higher crop yield depends on technically recommended NPK amount, application rates and time^2,22^. All types of fertilizers may not increase soil fertility at the same time because of it causes environmental pollution, decreases water quality, and has the most important adverse effects on human health^2^. So, if fertilizers are applied by keeping in mind their interaction with other nutrients as well as concerning soil chemical properties then more yield can be obtained per granular of fertilizers application^23,24^. The different methods for improving fertilizers use efficiencies have been recognized so far^25^. While, the idea of coated and slow-release fertilizers is also possible to adopt, for decreasing fertilizer rates (amount), and environmental pollution. The coating of sulfur and strains of *Thiobacillus* increase N, P and K use efficiencies due to long-term N availability in soil and sulfur reactions in the rhizosphere. Ghafoor et al.^2^ showed that coated fertilizers like sulfur and bioactive sulfur (*Thiobacillus*) increased phosphorus and potassium concentration in soil and wheat plant tissues resulting in higher wheat production under arid environmental conditions. The S element is also used as a fungicide and secondary nutrient, and neutralizes the alkalinity due to soil acidic properties^26^. Therefore, the monotypic urea cause more leaching and decrease soil fertility resulting in wheat yield reduction^27,28^. Neem-coated urea (NCU) has nitrification inhibition characteristics due to its chemical components like azadirachtin, melicians, de-acetyl, and salanin which showed coating percentage-based fertilizers treatments inhibition effects in soils^29^. Similarly, Manzoor et al.^22^ depicted that bacterial coated urea improved nutrients partial factor productivity and reduced N losses around the rhizosphere that resulting in higher cotton yield and its fiber qualities. There are limited studies available on the interaction of coated nitrogenous fertilizers on wheat yield and grain quality in an arid environment. There is a dire need to analyze coated fertilizers' adaptability under higher temperature conditions to enhance fertilizer use efficiencies by reducing nitrogen losses. The objective of the recent study is to examine the plant height, dry matter accumulation after anthesis (DMAA), contribution of dry matter accumulation after anthesis (CDMAA) towards grain yield, crop index, irrigation WUE (kg ha^−1^ mm^−1^), grain quality of wheat crop using different slow-release urea fertilizers (NCU, SCU and BSCU) in calcareous soils under arid environmental conditions.

## Materials and methods

An experimental trial was conducted during 2018–2019 in the higher temperature area of south Punjab (30.1598° N, 71.4502° E and altitude of 129 m) Pakistan. Further details like climatic conditions, pre-soil analysis are mentioned in Ghafoor et al.^2^.

### Research setup and crop management

Field research was conducted to determine the effects of various coated urea fertilizers on wheat crop yield, N, P and K use efficiencies, water relations, and crop index under arid environmental conditions. Different fertilizers product monotypic urea, sulfur coated urea, neem coated urea, bioactive sulfur coated urea, (sulfate of potash) SOP and (single super phosphate) SSP were used in experimental plots. Different levels of urea N 130, 117, 104 and 94 kg ha^−1^ were applied in three equal splits with SOP (62 kg ha^−1^) and SSP (114 kg ha^−1^) at the time of sowing as basal dose. Total of 250 mm irrigation was applied at the sowing, tillering and anthesis stages to fulfill the water requirements. Slow-release fertilizers including neem coated urea and simple urea was collected from Engro Fertilizers Limited Pakistan, while bioactive S-coated urea (BSCU) (*Thiobacillus*) by First Microbial BioTech. The S-coated urea is a product of Adfert UAE. Further, experiment design and crop management details were mentioned in Ghafoor et al.^2^.

### Grain NPK estimation

Wheat grain samples (0.1 g) were added to the digestion tube and in next step a concentrated 5 mL H_2_SO_4_ was also added. The tubes were incubated for 12 h at 25 °C temperature carefully. Then 1 mL of H_2_O_2_ (34%) was added in the digestion tubes. Further, the tubes were kept under 350 °C and waited till flame production. The duration of heating was set for 30 min. After that, the digestion tubes were cooled after the reheating procedure. Then, 1 mL of H_2_O_2_ were added and kept the tubes in the digestion block again. The same process was repeated many times until a colorless solution appeared. The material was put in the flask (50 mL) and the extract volume was set very cautiously. Finally, the filtrate N grain’s content was found by following Kjeldahl’s procedure.

5 mL extract and 10 mL Barton reagents were added in 50 mL flask and made up to mark by using distilled water. Standard was prepared by using potassium dihydrogen phosphate (KH_2_PO_4_) and volume made up to the mark by adding 10 mL Barton reagents and distilled water. The P grain concentration was measured by using a spectrophotometer at 420 nm wave.

Wheat grounded grain samples of 0.1 g and 5 mL concentrated H_2_SO_4_ were taken in the digestion tubes and incubated at 25 °C for 12 h. Then 1 mL of H_2_O_2_ 34% was added in the digestion tubes. Tubes were heated at 350 °C till fumes production. The heating of the samples was continued for 30 min, then the tubes were cooled and removed from the digestion block. After cooling, 1 mL of H_2_O_2_ was added and tubes were kept again in the block. The same process was repeated many times until colorless cooled digested material was obtained. Then the sample was added in 50 mL flask and made the volume. Extract filtration was done for K^+^ analysis. The samples were run on flame photometer and values were noted. The estimated values in ppm were compared with the standard curves. K (%) was determined by using the following formula.

1 $${\text{K }} ({\%})= \frac{{\text{ppm on graph }}\times {\text{dilution}}\times 100 }{{10}^{6}}$$

### Crop harvest index

The crop index (CI) was measured using the given formula^30^.

2 $${\text{Crop index}}=\frac{{\text{Grain yield}}}{{\text{Grain yield}}+{\text{Straw yield}}}$$

### Dry matter accumulation after anthesis (DMAA)

The DMAA is a difference between grain yield and dry matter translocation amount calculated, subtraction of dry biomass accumulation at anthesis from dry biomass accumulation at maturity phase.

3 $${\text{DMAA}}={\text{GY}}-{\text{DMT}}$$

### Contribution of dry matter accumulation after anthesis (CDMAA)

Contribution of dry matter accumulation after anthesis (CDMAA) is the ratio of dry matter accumulation after anthesis (DMAA) with final grain yield. It shows the translocation of assimilates shifted towards the grain after anthesis stage. It was calculated by given formula.

4 $${\text{CDMAA}}=\frac{{\text{DMAA}}}{{\text{GY}}}\times 100$$

### Yield and TDM measurements

The final yield and total dry matter after harvest was recorded from experimental plots separately, and on basis of dry biomass production converted into ton ha^−1^ (t ha^−1^).

### Statistical analysis

Statistical analysis was done by using the “R” statistical software (R version 3.6.1, https://www.r-project.org) by following Fisher’s two-way factorial ANOVA and Tukey’s HSD test at a 5% probability range to measure the average contrasts among tested treatments. Following R packages and library were used for data analysis (dplyr, agricolae, ggplot2, ggpubr, Hmisc, corrplot, Tukey’s HSD). We also primarily tested the research data for variance homogeneity and normality via Levene’s test and Shapiro–Wilk statistical test, correspondingly. The regression and correlation analysis completed for checked a clear depiction of different treatment result^31^.

### Ethics approval and consent to participate

We all declare that manuscript reporting studies do not involve any human participants, human data, or human tissue. So it is not applicable.

### Plant guidelines

All the plant experiments were in compliance with relevant institutional, national, and international guidelines and legislation.

## Results

### Plant height

Plant height data showed non- significant results among nitrogen sources, but found significant for N levels. Generally, maximum plant height recorded where recommended N 130 kg ha^−1^ was applied. The application of 130 kg N ha^−1^ with BSCU produced statistically maximum plant height (102.67 cm) while monotypic urea attained a lower plant height (99.60 cm), respectively. The application of urea 130 kg N ha^−1^ showed an increase of 22.02% in plant height than 94 kg N ha^−1^ application. The application of 117 kg N ha^−1^ NCU showed an increase of 9.37% in plant height compared with 94 kg N ha^−1^. The applications of 94 kg N ha^−1^ with SCU and BSCU produced the plant heights of 79.33 and 82.67 cm, respectively (Table 1).

**Table 1:** Effect of different slow release fertilizers and N rates on plant height (cm), DMAA (kg ha^−1^, CDMAA (%) and grain NPK%

Treatments	Plant height (cm)	DMAA (kg ha^−1^)	CDMAA (%)	Grain N (%)	Grain P (%)	Grain K (%)
UreaN0	99.60 abc	1289 e	31.99 d	1.97 d	0.58 d	0.93 c
UreaN1	97.67 c	965 i	28.04 f	1.21 g	0.47 g	0.73 f
UreaN2	86.00 ef	615 m	25.40 g	0.96 ij	0.33 j	0.47 ij
UreaN3	77.67 h	315 p	17.01 i	0.77 k	0.19 m	0.35 k
NCUN0	97.00 c	1478 bc	35.45 c	2.51 c	0.59 c	0.95 bc
NCUN1	98.21 bc	1110 g	31.66 d	1.45 f	0.53 f	0.77 ef
NCUN2	89.00 de	687 l	27.24 f	1.08 h	0.35 i	0.50 i
NCUN3	80.67 gh	404 o	19.99 h	0.88 j	0.23 l	0.38 k
SCUN0	102.00 ab	1744 ab	39.39 b	2.99 b	0.62 b	0.97 b
SCUN1	97.00 c	1250 f	34.63 c	1.77 e	0.54 ef	0.78 e
SCUN2	91.00 d	943 j	30.14 e	1.29 g	0.36 i	0.56 h
SCUN3	79.33 gh	562 n	21.28 h	1.04 hi	0.24 kl	0.44 j
BSCUN0	102.67 a	1989 a	44.63 a	3.54 a	0.66 a	1.07 a
BSCUN1	98.33 bc	1472 d	38.20 b	2.05 d	0.55 e	0.84 d
BSCUN2	92.00 d	1085 h	32.47 d	1.45 f	0.40 h	0.60 g
BSCUN3	82.67 fg	740 kl	27.35 f	1.22 g	0.25 k	0.48 ij
Interactions sources × N levels		**	**	**	**	**

N0 130 kg N ha^−1^, N1 117 kg N ha^−1^, N2 104 kg N ha^−1^, N3 94 kg N ha^−1^, *BSCU* bioactive sulfur coated urea, *SCU* sulfur coated urea, *NCU* neem coated urea, *CDMAA* dry matter accumulation contributed amount after anthesis, *DMAA* accumulated dry matter after anthesis stage.

### Translocation of dry matter between anthesis to maturity in vegetative plant’s organ

The results showed that optimum N rates application with effective sources showed significant results under arid environmental conditions. The BSCU 130 kg N ha^−1^ produced a higher DMAA (1989 kg ha^−1^) while the minimum values of the DMAA (315 kg ha^−1^) was found with the application of Urea at the rate of 94 kg ha^−1^. The SCU with 130 kg N ha^−1^ recorded a decrease of 14% as compared to BSCU with the same N level. The urea with the application of N 130 kg ha^−1^ showed a values of 1289 kg ha^−1^ DMAA. The NCU with recommended N showed an increase of 13.11% as compared to urea with the same N rate. The urea 104 kg N ha^−1^ showed a decrease of 11.71% compared with NCU 104 kg N ha^−1^ application (Table 1). Overall, research data showed that the assimilation of dry matter after anthesis was found maximum with the application of recommended N (130 kg ha^−1^) and coated fertilizers than monotypic urea and lower N rates. The distribution after anthesis to develop grain phase remained maximum with the application of higher N rates and coated fertilizers after anthesis growth phase in wheat plant under arid environment. It was seen that CDMAA showed significant results both for different N rates and urea products. The highest value of CDMAA (44.63%)observed where BSCU with 130 kg N ha^−1^ was applied. While, the lower values and a decreases of 23.05% and 36.22% was recorded with the application of monotypic urea 117 kg N ha^−1^ as compared to SCU and BSCU with a same N rate application, respectively. The minimum value of CDMAA17.01%shown by urea with 94 kg N ha^−1^ rate application. The SCU and BSCU with the application of N rate 94 kg ha^−1^ showed the values of CDMAA 19.99% and 27.35%, respectively. The NCU with application of 117 kg N ha^−1^ showed an increase of 11.42% as compared to monotypic urea with same N level (Table 1).

### Grain nutrients N, P and K concentrations

Grain nutritional qualities are improved by providing optimum N application and slow release fertilizer. The BSCU with N 130 kg ha^−1^ application depicted maximum grain N, P and K% concentrations. The SCU with N 130 kg ha^−1^ showed a lower grain N% (18.4%) than BSCU. The NCU with the application of N 117 kg ha^−1^ showed an increase of 16.5% in grain N% as compared to ordinary urea with the same N rate. The minimum grain N% recorded with the application of monotypic urea (94 kg N ha^−1^). Maximum grain N (97.90 kg ha^−1^) was recorded by N 130 kg ha^−1^ BSCU application while minimum grain N (14.24 kg ha^−1^) was noted where urea (94 kg N ha^−1^) applied. The NCU with N 130 kg ha^−1^ showed an increase of 8.15% as compared to urea with the same N rate under arid climatic conditions (Table 1).

The maximum grain P (0.66% and 29.57 kg ha^−1^) was observed with the application of BSCU and optimum N 130 kg ha^−1^, correspondingly. The monotypic urea with N 130 kg ha^−1^ application showed low P (0.58% and 23.23 kg ha^−1^) in grains. The SCU with 117 kg N ha^−1^ showed an (kg ha^−1^) increase of 15.94% in grain P as compared to monotypic urea with a similar N application. The minimum grain P 3.57 kg ha^−1^ and P 0.19% recorded from monotypic urea with 94 kg N ha^−1^. The BSCU with N 104 kg ha^−1^ showed a grain P % increase of 8.40% as compared to SCU with equal N application.

**Figure 1:**
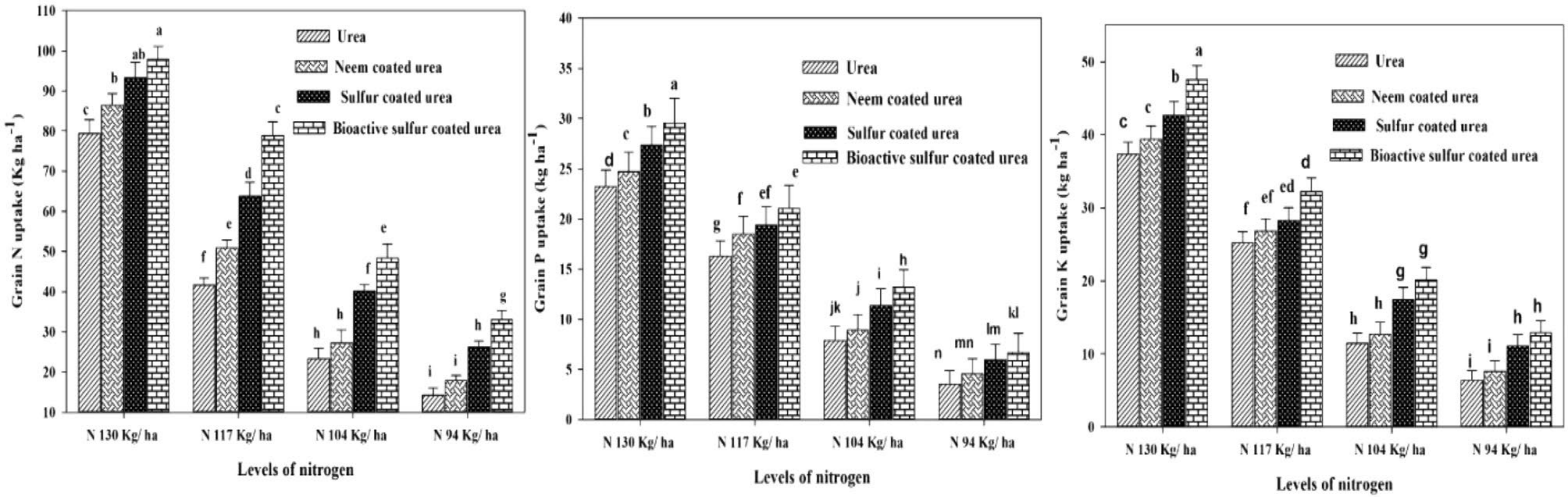
Effect of different slow-release fertilizers and N rates on grain NPK uptake (kg ha^−1^) under arid environmental conditions.

Results showed that BSCU with N 130 kg ha^−1^ produced maximum grain K (kg ha^−1^) 47.56 and 1.07% as compared to other treatments. Research depicted the positive relationship between slow-release urea N levels, and soil and grain NPK concentrations (Fig. 1). Different sources monotypic urea, NCU and SCU showed a decrease of 15.11%, 12.67%, and 9.59% in grain K% as compared to BSCU with the same N level respectively. The NCU with N 117 kg ha^−1^ application showed an increase in grain K% as compared to ordinary urea. Similarly, the BSCU showed a grain K% increase of 6.37% as compared to SCU when 117 kg N ha^−1^ was applied. The minimum grain K (0.35% and 6.39 kg ha^−1^) were obtained with urea 94 kg N ha^−1^ application. The BSCU with 94 kg N ha^−1^ showed a grain K% increase of 7.69% as compared to SCU with a similar N rate under an arid environment. Research findings depicted a positive interaction between slow-release fertilizers and their rates with growth attributes like TDM, and grain production (Fig. 3).

### Yield components of wheat crop under an arid environment

**Figure 2:**
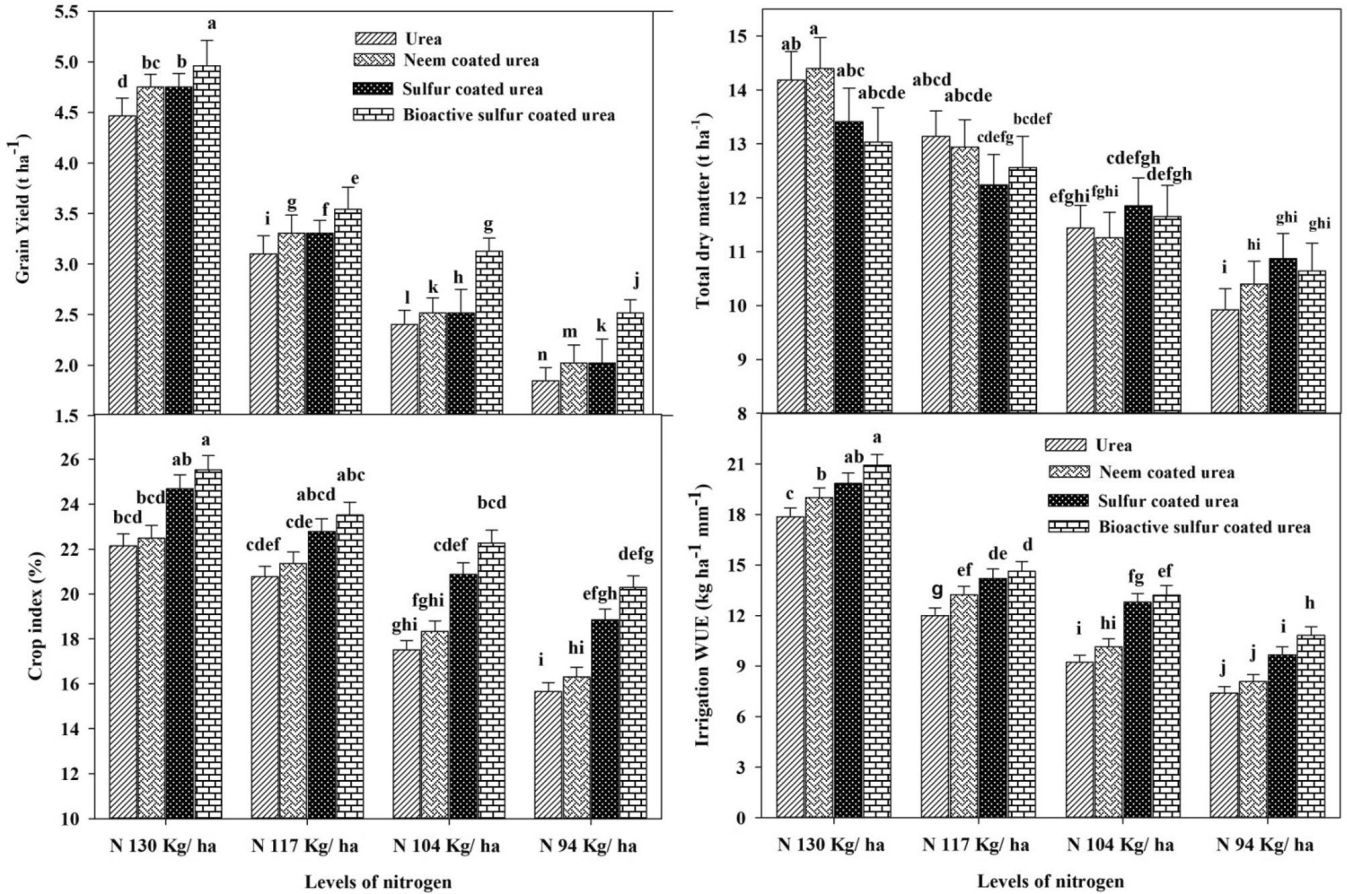
Effect of different slow release fertilizers and N rates on grain yield (t ha^−1^), total dry matter (t ha^−1^), crop index% and irrigation water use efficiency (kg ha^−1^ mm^−1^).

There is a high TDM of 14.40 t ha^−1^ obtained by NCU with 130 kg N ha^−1^ applied while the minimum TDM of 9.92 t ha^−1^ was recorded by 94 kg N ha^−1^ urea application. Results showed that the BSCU, SCU, NCU and monotypic urea with N 130 kg ha^−1^ application showed statistically same TDM in calcareous soils under arid environmental conditions. Further results revealed that urea with 117 kg N ha^−1^ showed an increase (1.52%) as compared to NCU with a similar N rate. Furthermore, the BSCU and SCU with 117 kg N ha^−1^ application showed statistically at par results under arid environments (Fig. 2).

The results of current research showed that mineral-coated fertilizers increased grain yield as compared to neem-coated and monotypic urea. The BSCU 130 kg N ha^−1^ produced the highest grain yield (5.23 t ha^−1^). Similarly, other coated urea products like NCU and SCU depicted excellent results in term of growth and yield related parameters than granular urea. Results revealed that the NCU 130 kg N ha^−1^ showed an increase of 6.05% than urea, while BSCU showed an increase of 4.12% than NCU with similar N rates. It was seen that BSCU 117 kg N ha^−1^ showed an increase of 2.93 and 9.58% as compared with SCU and NCU for similar N rates, respectively. Further, the NCU and BSCU with the application of 117 and 104 kg N ha^−1^ showed statistically similar results. While the minimum grain yield (1.85 t ha^−1^) was observed in plots where urea 94 kg N ha^−1^ was applied only (Fig. 2).

### Irrigation WUE (kg ha^−1^ mm^−1^)

**Figure 3:**
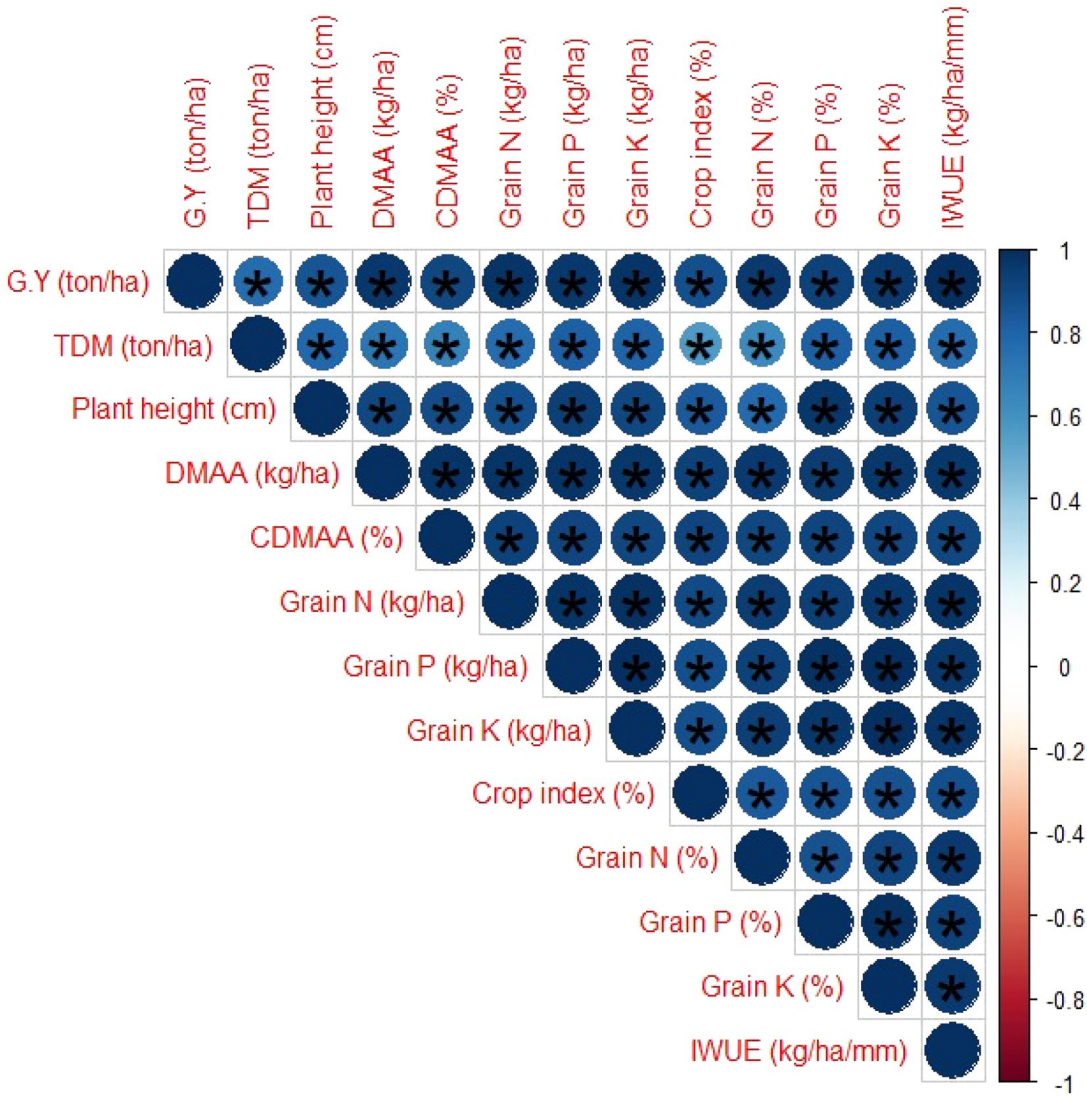
Correlation of various soil nutrients with yield, crop index (%), irrigation water use efficiency (kg ha^−1^ mm^−1^) at various N rates and slow release fertilizers. The circle’s areas depict the absolute result of corresponding correlation coefficients tested at *0.01 significance level. Light and dark blue colors depicted the minimum and maximum values. Star (*) and non-star presented significance and non-significance. *GY* grain yield, *TDM* total dry matter, *Grain N* grain nitrogen, *Grain P* grain phosphorus, *Grain K* grain potassium, *Irrigation WUE* irrigation water use efficiency, *DMAA* dry matter accumulation after anthesis, *CDMAA* contribution of dry matter accumulation after anthesis to wheat grains.

Coated fertilizers also significantly improved irrigation WUE of wheat crops under arid environmental conditions. Results showed that elemental S coating of urea also improved the water uptake capacity of plants as compared with monotypic and neem oil coating urea (Fig. 2). The BSCU 130 kg N ha^−1^ showed the highest irrigation WUE of 20.92 (kg ha^−1^ mm^−1^) and an increase of 5.15% than SCU. Similarly, the NCU and SCU with 130 kg N ha^−1^ depicted irrigation WUE 19.01 and 19.84 (kg ha^−1^ mm^−1^) respectively. The SCU and BSCU N 117 kg ha^−1^ application also showed statistically similar results. While minimum irrigation WUE of 7.39 (kg ha^−1^ mm^−1^) was observed by urea 94 kg N ha^−1^ application. Availability of nutrients in soil depicted a positive interaction with irrigation WUE (kg ha^−1^ mm^−1^). Optimum N rate 130 kg ha^−1^ application showed strong interaction with irrigation WUE (kg ha^−1^ mm^−1^) as compared with lower levels like 104 and 94 kg N ha^−1^ application (Fig. 3).

### Crop index (%)

Crop index (%) also showed statistically highly significant with the application of coated urea and nitrogen increment. Maximum and minimum crop index of 25.52 and 15.66% were shown by BSCU and monotypic urea with 130 and 94 kg N ha^−1^ applications, respectively. Mineral coated urea BSCU and SCU by the application of N 130 and 117 kg ha^−1^ indicated statistically similar results for crop index (%). Monotypic urea 130 kg N ha^−1^ depicted a decrease of 15.21% and 11.45% than BSCU and SCU with similar N rates, respectively (Fig. 2). Further results declare that NCU 94 kg N ha^−1^ showed a higher (3.94%) crop index as compared with urea by the application of same N level. Similarly, the BSCU 104 kg N ha^−1^ application showed an increase of 6.18% in crop index than SCU with a similar N rate. Furthermore, the NCU and monotypic urea showed statistically similar results when N 130 kg ha^−1^ was applied. Current research showed a positive interaction between coated fertilizers and their levels with crop index (%) (Fig. 3).

## Discussion

Current research showed that wheat responds differently to different coated fertilizers under the arid region in calcareous soils. There is a positive interaction between coated urea products concerning N increments. In the current research, the BSCU with recommended N rate of 130 kg ha^−1^ performed better than other sources and rates (Fig. 3). Experimental findings showed that optimum N application increased plants height significantly, but different coated fertilizers were not found effective for plant height. The results were accord with Ullah et al.^32^ those showed that higher nitrogen increased the plant height of wheat crop. Research showed that recommended N (130 kg N ha^−1^) produced a maximum dry biomass assimilation after anthesis stage, these findings are in line with previous research of Zhang et al.^27^. The results are also accord with Li et al.^33^ which showed that optimum N and water improved biomass accumulation after anthesis in wheat crop.

Coated fertilizers enhanced NPK concentrations in soil and plants and nutrient use efficiencies of wheat crop under semiarid conditions^34^. The positive effects of NCU were observed over monotypic urea due to nitrification inhibition properties of neem oil. Similarly, Ali et al.^35^ also showed that NCU reduced soil N as compared with ordinary granular urea. An application of 100% NCU increased soil available K, N and P concentrations 490.63, 188.40 and 18.10 kg ha^−1^ in pearl millet crop^36^ under arid climatic conditions.

Different N rates and coated fertilizers enhanced grain qualities and grain yield. The optimum N application increased grain nutrients concentrations. In the current experiment, the mineral-coated urea like SCU and BSCU enhanced grain nutrients NPK concentrations and their uptake as compared with monotypic urea. The subsurface application of coated N fertilizers increased grain N% up to 57% than uncoated N fertilizers with surface application^34^. Shivay et al.^28^ depicted that 5% SCU increased grain N% and uptake up to 2.6% and 98.6 (kg ha^−1^) than other treatments, respectively. It was due to that slow release fertilizers increased duration of soil N availability which was initially up taken by vegetative parts and then translocate to cereals grains^22,27,35,37^. Furthermore, Ali et al.^35^ presented that neem oil coated urea (NOCU) enhanced the plant N concentrations at optimum N application over monotypic urea. The consistent availability of soil N concentrations may enhance grain N concentration and yield to an extent and then enhanced residual N in straw at maturity period^38^. Generally, pot research confirmed that the optimum rate of NPK increased N uptake, physiological efficiencies of shoot and grain at maturity period^39^. Enhanced efficiency of fertilizers with 50% of total N applied at the booting stage had highly significant association with grain quality that proved split application benefits for chemical qualities of wheat^40^. The findings of previous research also showed that polymer coated controlled release urea increased soil N, P and K availability and such synergistic effects led to higher grain yield, N uptake and its recovery (%) in clay loam soils Nezami et al.^41^ and proved strong support for the current research findings.

Wheat response to coated fertilizers and different N increments in low organic matter soils is different due to the higher temperature in the region being arid climate zone while improvement in fertilizers manufacturing technology is required to enhance grain yield to ensure the food requirements. The current experiment showed that mineral coated fertilizers and bacterial coated fertilizers increased the grain yield. Different N levels and sources of coated urea showed significant results for irrigation water use efficiency in wheat crop in an arid environment. Awaad et al.^42^ recommended that slow-release N fertilizers (100 kg N fed^−1^) with followed by drip irrigation at 100% field capacity increased water productivity, wheat yield and nitrogen use efficiencies under semi-arid climatic conditions. The soil N was released rapidly at an early crop stage, and making it highly susceptible for N deficiency in plant during later crop growth stages and proved unfavorable to improve water use efficiency, grain yield and biomass of maize crop^43^. Optimum N application enhanced biomass production that also enhanced the requirement for roots to absorb maximum water from the deeper soil layer. The combination of slow release and monotypic N fertilizers enhanced the maize root length and weight densities at dent stage in the deeper soil layers^44^. Hu et al.^45^ found that controlled release urea increased deeper root growth, and deeper soils has more soil water storage making it easy for plants to uptake water to fulfill its requirement at maize dent growth phase. Further, they concluded that a combination of slow and monotypic fertilizers proved good technique for improving water use efficiencies and grain yield under semiarid and arid climatic conditions.

Slow release N fertilizers slow down the nutrient release from the fertilizer granular which not only increased soil fertility profile but also maintained the nutrients supply to plants at critical stages^2,22,35^. Our results showed that BSCU and SCU showed more crop index % than uncoated and neem coated urea. This might be due to slow release fertilizers balanced the nutrients in soil and more availability in plants rhizosphere^2,37,41^. The S has acidic properties and neutralized the soil's alkalinity^2^. Basically, microbial activity with the availability of S reduced soil pH in the rhizosphere resulted in increased availability duration of nutrients in the calcareous soils. Further *Thiobacillus* bacteria strains also enhanced S oxidation process in soil and reduced soil pH which making it easier for plants to uptake N more efficiently^46^. Slow release fertilizers enhanced the availability of P and K in soils, so uptake of P and K are also improved and translocate to grain. Khan et al.^47^ showed that K fertilization required equally P and N nutrients to obtain the desire yield and yield gaps. Furthermore, overall the findings of crop index % are also agreed with Saleem et al.^48^ who found that coated N fertilizers recommended NP (10% KFeO_2_ nano-coated DAP + urea) increased crop index up to 0.37% as compared to other treatments combinations under semiarid environmental conditions. It is evident from the findings and discussion that slow release nitrogenous fertilizer has the potential to improve the growth, yield of wheat crop under arid environmental conditions by reducing the N losses.

## Conclusion

Slow-release nitrogen fertilizers have positive and enhanced effects on wheat crop growth and yield under arid environmental conditions. While different nitrogen levels showed significant effects on plant height as well. The current research depicted the positive relation of slow-release fertilizers with crop index, DMAA, CDMAA, irrigation water use efficiency, grain yield and grain NPK. Different slow release fertilizers and nitrogen rates also significantly affected the DMAA, CDMAA, irrigation water use efficiency and grain yield. A significant increase in grain NPK% and uptake (kg ha^−1^) was recorded by 130 kg N ha^−1^ BSCU. Further, it was observed that more irrigation WUE (20.92 kg ha^−1^ mm^−1^) was obtained by BSCU 130 kg ha^−1^ under arid climatic conditions. Among the N application, the 90 and 80% N levels with bioactive sulfur coated urea also showed significant results than neem coated and monotypic urea. So, SCU and BSCU with 80 and 90% N levels may also be recommended for better crop and finally sustainable wheat production under arid environmental conditions. Furthermore, multi-location and multi-years research like the ecosystem services modeling under various cropping systems and climate change scenarios may also suggested for future studies.

## Data Availability

The datasets generated and/or analyzed during the current study are not publicly available due to institutional restricted policy but are available from the corresponding author on reasonable request.

## Code availability

The codes used and/or analyzed during the current study are available from the corresponding author on reasonable request.
